# Centrosomal protein 290 is a novel prognostic indicator that modulates liver cancer cell ferroptosis via the *Nrf2* pathway

**DOI:** 10.18632/aging.203946

**Published:** 2022-03-10

**Authors:** Yiru Shan, Guang Yang, Qiuhong Lu, Xiangyu Hu, Dongwei Qi, Yehan Zhou, Yin Xiao, Li Cao, Fuhua Tian, Qi Pan

**Affiliations:** 1 Department of Oncology, Jiulongpo People’s Hospital of Chongqing, Chongqing, P.R. China; 2 Department of Urology Surgery, The First Affiliated Hospital of Chongqing Medical University, Chongqing, P.R. China; 3 Department of Orthopaedics, Jiulongpo People’s Hospital of Chongqing, Chongqing, P.R. China; 4 Department of Dermatology, Chongqing Hospital of Traditional Chinese Medicine, Chongqing, P.R. China; 5 Department of Pathology, Sichuan Cancer Hospital and Institute, Sichuan Cancer Center, School of Medicine, University of Electronic Science and Technology of China, Chengdu, P.R. China; 6 Department of Patient Service Center, Jiulongpo People’s Hospital of Chongqing, Chongqing, P.R. China

**Keywords:** ferroptosis, hepatocellular carcinoma, CEP290, Nrf2 signaling pathway

## Abstract

Ferroptosis is an iron-dependent form of cell death. In spite of its significance in pathogenesis and disease progression, ferroptotic signal transduction in HBV-HCC has not been fully explained. Here, four HCC open-source datasets were downloaded from the GEO repository. Cox regression and LASSO models were established to prioritize novel prognostic candidate biomarkers, and the results were verified *in vitro* and *in vivo*. We identified 633 common DEGs in both of the bulk RNA-Seq expression profiles. Next, based upon the TCGA-LIHC cohort, a prognostic signature consisting of nine genes was extracted from 633 shared DEGs, and the specificity and sensitivity of the signature were evaluated in both training and validation datasets. This signature showed that the high-risk group had a worse prognosis than the low-risk group. CEP290 was discovered among the prognostic signature genes, and its expression notably correlated with survival, AFP level, TNM stage and vascular invasion. We confirmed expression of CEP290 in eight pairs of HCC tissues and diverse liver cancer cell lines. CEP290 knockdown reduced proliferation, migration and invasion in Hep3B liver cancer cells while Fe2+ and malondialdehyde levels were elevated. Mechanically, co-immunoprecipitation showed an interaction between CEP290 and Nrf2 proteins, and biological phenotypes of Hep3B cells under CEP290 interference were rescued by Nrf2 activator. Furthermore, CEP290 silencing considerably blocked protein expression of Nrf2 pathway members. Finally, suppression of CEP290 effectively inhibited tumor growth *in vivo*. The above results shed light on the important role of CEP290 in ferroptosis and present an important implication for HCC progression.

## INTRODUCTION

Hepatocellular carcinoma (HCC) is the 4^th^ most common cause of cancer-associated mortality worldwide [1], and hepatitis B virus (HBV) infection accounts for the majority of cases [2]. In developing countries such as China, the HCC incidence rate has been increasing continuously [3, 4]. At present, accurate biomarkers for HCC prognosis prediction have not been identified. Therefore, identifying predictive biomarkers is critical for the assessing the HCC survival rate [5, 6].

Great progress has been made in the analysis of transcriptomes with high-throughput sequencing technologies, and these studies have revealed detailed gene expression patterns in diverse cancers [7]. Bioinformatic analysis of gene expression profiles facilitates biomarker identification. For example, centrosomal protein 131 (*CEP131*) was recognized as a novel substrate of *PLK4* that facilitated centrosome amplification and colon cancer development [8]; overexpression of centrosomal protein 70 (*CEP70*) stimulated the growth of pancreatic cancer cells by inducing abnormal centrosomes and disorganized microtubules [9]. These studies revealed that centrosomal proteins could be potential biomarkers and therapeutic targets and played critical roles in diverse cancers.

Ferroptosis is a form of iron-dependent cell death, which is different from autophagy, necrosis and apoptosis [10]. Due to iron-dependent accumulation of lipid peroxidation, ferroptosis is regulated by a specific set of proteins involved in many cancer signaling pathways related to iron metabolism [11, 12]. *Nrf2*, a core gene for the oxidant stress response, induces the activation of its downstream genes that function against tumor ferroptosis [13]. Moreover, ferroptosis can also be triggered by the aberration of the glutathione (GSH)-glutathione peroxidase 4 (*GPX4*) antioxidant systems, indicating that *GPX4* is the vital link of antiperoxidant defense [14, 15]. However, the potential ferroptosis-related regulatory mechanism remains unknown.

In the present work, we demonstrate that centrosomal protein 290 (*CEP290*), which regulates cancer cell ferroptosis, growth, migration and invasion through the *Nrf2* signaling pathway, is a reliable biomarker for prognosis prediction. Bioinformatics analysis in combination with *in vivo* and *in vitro* experiments represents a more effective approach for exploring the molecular mechanism of HCC.

## MATERIALS AND METHODS

### HCC cases

For this study, we acquired eight HCC specimens and paired normal liver specimens from HBV-associated HCC patients who underwent surgical treatment at Sichuan Cancer Hospital. The Research Ethics Committee of Sichuan Cancer Hospital approved the research design. All patients signed informed consent for participation. After collection, each specimen was frozen immediately in liquid nitrogen until processed for subsequent assays.

### HCC RNA-Seq dataset acquisition

To identify differentially expressed genes (DEGs) between HBV-associated HCC specimens and non-carcinoma liver specimens (NL), this work made use of two open-source datasets, GSE22058 (n=192) [16] and GSE54238 (n=23) [17], from the GEO database using GPL6793 together with the GPL16955 platform. One 10×Genomics single-cell RNA sequencing dataset (GSE103867) including resected tissue from a primary HCC patient and HBV-negative liver cancer Huh1 and Huh7 cell lines [18] was downloaded from the Gene Expression Omnibus (GEO) repository.

### DEG recognition

After mean background correction of multi chips, we normalized quantiles and calculated the expression levels of the microarray matrix by Affymetrix to obtain specific gene expression data. In addition, we adopted Limma and Bayesian statistics to construct a linear model. This study selected DEGs from HCC specimens upon the Log2 (fold change, FC) |> 0.6 and *p* <0.05 thresholds.

### Annotation of biological function

We used Gene Ontology (GO) analysis as the primary reference for the annotation of genes or gene products or for the interpretation of high-throughput genomic and transcriptomic analysis results [19]. We also used DAVID database (https://david.ncifcrf.gov/) for mapping a user-defined gene for related biological annotations, which plays an important role in the successful analysis of genes post-HTS [20]. The present work adopted DAVID for GO analysis to examine DEG effects. *p*<0.05 stood for statistical significance.

### HCC prognosis nomogram construction

The survival rate was calculated based on Kaplan–Meier (KM) curve analysis, and significant differences across diverse survival curves were determined by the log-rank test. In addition, the Cox proportional hazards model was utilized for univariate and multivariate analyses. Later, the LASSO-Cox method was adopted to reduce the dimensionality, and we selected significant HCC prognostic genes to construct a prognostic model based on Cox regression [21]. The LASSO approach was adopted to establish and validate a nomogram [22]. Afterwards, we drew receiver operating characteristic (ROC) curves and then determined the area under the curve (AUC) values to analyze the specificity and sensitivity of our model [23].

### Immunohistochemical (IHC) staining

Eight HBV-HCC and matched normal specimens were dehydrated, paraffin embedded, sliced into 4-μm sections, deparaffinized with xylene and rehydrated with a series of ethanol solutions at room temperature. Then, antigen recovery was performed using sodium citrate, endogenous peroxidase activity was blocked using 3% H_2_O_2_, and specimens were blocked using 5% bovine serum albumin (BSA) at room temperature for 30 min. Subsequently, specimens were incubated with anti-*CEP290* primary antibody (No. 22490-1-AP; Proteintech, China) for 12 h at 4° C, followed by an additional 2 h of incubation with HRP-labeled secondary antibodies at room temperature. Color development was conducted using a Cell and Tissue Staining HRP-DAB kit (Beyond) according to manufacturer’s protocols. Images were acquired using an Orthophoto microscope (×100). IHC results were semi-quantitatively analyzed by using ‘image-pro_plus’ software. IOD values and region area were measured by selecting both control and target regions. We determined the average density (IOD/area) within both regions.

### Cell culture and treatment

Huh7 and Hep3B liver cancer cells, together with immortalized non-cancerous THLE-2 cells, were provided by the Cell Bank of the Chinese Academy of Sciences. All cells were cultured in RPMI-1640 medium (Gibco, USA) supplemented with 10% fetal bovine serum (FBS; Gibco, USA) and 1% penicillin-streptomycin at 37° C with 5% CO_2_.

For inhibition of *CEP290* expression, *CEP290*-siRNAs and the relevant negative control (NC) siRNAs were acquired from RIBOBIO (Guangzhou, China). siRNA sequences used in the present work are shown below: si-*CEP290* #1: ID stB0013686A; si-*CEP290* #2: ID stB0013686B. Cells (1×10^5^/well) were plated into 6-well plates and cultured until they reached 70%–80% confluence. Lipofectamine 2000 (Invitrogen, USA) was used to transfect siRNA into cells at a final concentration of 100 μM according to manufacturer’s protocols. Target gene levels were determined at 48 h post-transfection.

### RNA isolation and qRT–PCR

The RNAiso Plus kit (Takara, USA) was used to extract total RNA according to manufacturer’s protocols. Complementary DNA was prepared from the extracted RNA by a PrimeScriptTM RT Reagent kit with gDNA Eraser (Takara, USA) by reverse transcription under the following conditions: 15 min at 37° C, 5 s at 85° C and 5 min at 4° C. The CFX96 Touch Real-Time PCR system (Bio-Rad, USA) and SYBR Premix Ex Taq II (Takara, USA) were used for qPCR under the following conditions: 30 s at 95° C; followed by 5 s at 95° C and 30 s at 60° C for 40 cycles. The 2-ΔΔCq approach was employed to quantify relative gene expression levels, with *GAPDH* as the endogenous reference. The primers used in the present work are shown below: for *GAPDH,* 5′-CTTTGGTATCGTGGAAGGACTC-3′ (forward), 5′-GTAGAGGCAGGGATGATGTTCT-3′ (reverse); for *CEP290,* 5′- GATGCTCACCGAACAAGTAGAAC-3′ (forward), 5′- ATGAGTCTGTTGAGAAAGGGTTG-3′ (reverse). All the reactions were performed in triplicate.

### Cell proliferation analysis and colony formation experiment

Cell proliferation was determined with Cell Counting Kit-8 (CCK8, Dojindo, Japan) according to manufacturer’s instructions. In brief, cells (4×10^3^/well) were plated in 96-well plates and cultured for 0, 24, 48 and 72 h. Afterwards, CCK-8 solution was added to each well and incubated for 1.5 h in the dark. Thereafter, we determined the living cell count by measuring the absorbance (OD) at 450 nm.

Cells were seeded into 6-well plates (600 cells/well for the si-*CEP290* cell model and the si-*CEP290* + Oltipraz cell model) using three replicate wells for each group, and were cultured in 5% CO_2_ incubator at 37° C. After one week, the cells were fixed with 4% paraformaldehyde for 30 minutes and incubated with crystal violet. After rinsed with PBS three times, the clones were recorded and the number of clones was calculated.

### Scratch assay

Migration of the treated or untreated Hep3B cells was evaluated with an *in vitro* scratch assay. In brief, cells were grown in 6-well plates until they reached 100% density. A sterile pipette tip was utilized to make a wound in the cell monolayer, detached cells were removed by rinsing three times with PBS, and cells were further cultured in serum-free medium for 24 h. Images were taken at 0 and 24 h.

### Transwell assay

The migration and invasion of Hep3B cells were examined by Transwell assay. The treated or untreated Hep3B cells were resuspended in serum-free medium. For measuring cell migration, we first added 100 μL as-prepared cell suspension into the Matrigel (BD Biosciences)-coated upper chamber, and 600 μL medium that contained 10% FBS was added to the lower chamber. Then, 4×10^4^ cells were added into the upper chamber and fixed using 4% paraformaldehyde for 15 min. Twenty-four hours later, we removed the cells from the chamber. Then, the cells were stained with crystal violet (0.1%) for a period of 10 min. Then, we selected cells located in the inner layer and randomly chose 3 fields of view (FOVs) in each sample to determine the number of penetrating cells.

### Iron assay

Relative iron levels within cell lysates were assessed using an iron assay kit (No. DIFE-250; BioAssay Systems, USA) according to manufacturer’s instructions.

### Lipid peroxidation analysis

A lipid peroxidation assay kit (No. A003-1-2; Nanjing Jiancheng College of Biotechnology, China) was used to assess malondialdehyde (MDA) content within cell lysates according to manufacturer’s instructions.

### Western blotting (WB) analysis

Total proteins were extracted from cells, and protein levels were measured using a BCA protein assay kit (Beyotime, Shanghai, China). Proteins were separated by 8% SDS-PAGE and transferred to PVDF membranes. After blocking in 5% skimmed milk, the membranes were incubated with primary antibodies at 4° C overnight. Next day, the membranes were rinsed with TBST three times, incubated for 1 h with secondary antibody at ambient temperature, rinsed with TBST three times and visualized by ECL (Wanleibio, Shenyang, China). The following antibodies *CEP290* (ab85728, Abcam, USA), *Nrf2*, *NQO1*, *HO-1* (Proteintech, Wuhan, China), *FTH1* (Cell Signaling Technology, USA) and β-actin (DianyinBio, Shanghai, China) were used in this study.

### Xenograft assay in nude mice

Athymic nude (BALB/c) mice were acquired from Chongqing Hospital of Traditional Chinese Medicine. Six-week-old male mice were injected with Hep3B cells and *CEP290*-deficient Hep3B (*CEP290*-KO) cells (2×10^6^ cells) in the right flanks into the subdermal space (n=5 per group). Tumors were measured with a caliper on days 0, 4, 8 and 14, and the volume was estimated according to the formula: volume = tumor length in mm × width^2^ in mm × 0.5236. Fourteen days post-injection, all animals were euthanized and the tumor tissues were collected. All animal procedures were carried out following the Guidelines for Animal Experiments released by the Chinese government.

### Statistical analysis

Data were displayed as mean ± SD. GraphPad Prism analysis software was used for statistical analyses. One-way ANOVA and t-test were performed to assess differences of 2 groups, while those across multiple groups were analyzed through LSD-*t* test and one-way ANOVA. * *p*<0.05; ** *p*<0.01; # *p*<0.05; ## *p*<0.01.

### Data availability

The data that support the findings of this study are available from the corresponding author upon reasonable request.

## RESULTS

### Shared DEGs identified in the 2 HCC datasets

**Figure 1:**
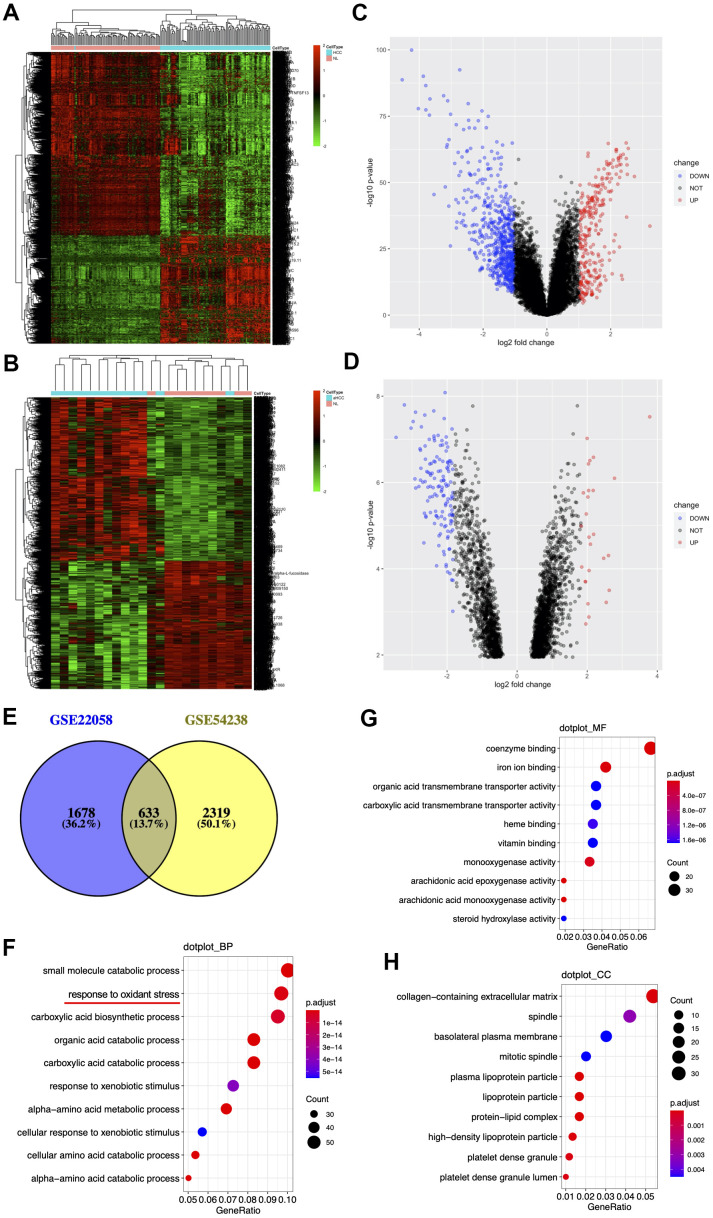
**Identification and enrichment of DEGs from the GSE54238 and GSE22058 datasets.** (**A**, **B**) DEGs hierarchical clustering. Data are the log_2_ HCC-to-normal intensity ratio. Green and red represent significantly downregulated and upregulated genes (*p* <0.05), respectively. aHCC, advanced hepatocellular carcinoma. (**C**, **D**) Volcano plot representing the distribution of DEGs. (**E**) DEGs shared in two datasets. (**F**–**H**) Ten significant GO terms.

Two open-source HBV-related HCC datasets GSE54238 (n=23) and GSE22058 (n=192) were downloaded from the GEO repository. DEG expression data were investigated using the Limma R package in both datasets. We identified 2,311 DEGs between 96 HCC specimens and paired non-cancerous specimens from the GSE22058 dataset with the threshold set at *p* < 0.05 and | Log2 (FC) | > 0.6, with 859 genes upregulated and 1,452 downregulated (Figure 1A, 1B). 2,952 DEGs were identified from the GSE54238 dataset when 13 HCC specimens were compared with 10 non-cancerous specimens, with 1,658 genes upregulated and 1,294 downregulated (Figure 1C, 1D). There were 633 shared DEGs between the two datasets (Figure 1E).

To better understand the biological roles of 633 shared DEGs, we used the ‘clusterProfiler’ [24] function in the R package for functional annotations. GO terms can be divided into three categories, biological processes (BPs), molecular functions (MFs) and cellular components (CCs). For BPs, DEGs were mostly associated with ‘small molecule catabolic process’, ‘response to oxidative stress’, and ‘carboxylic acid biosynthetic process’ (Figure 1F). As for MF, DEGs were mostly related to ‘coenzyme binding’, ‘iron ion binding’ and ‘organic acid transmembrane transporter activity’ (Figure 1G). Concerning CC, those DEGs were mostly associated with ‘collagen-containing extracellular matrix’, ‘spindle’ and ‘basolateral plasma membrane’ (Figure 1H).

### HCC prognosis prediction model

**Figure 2:**
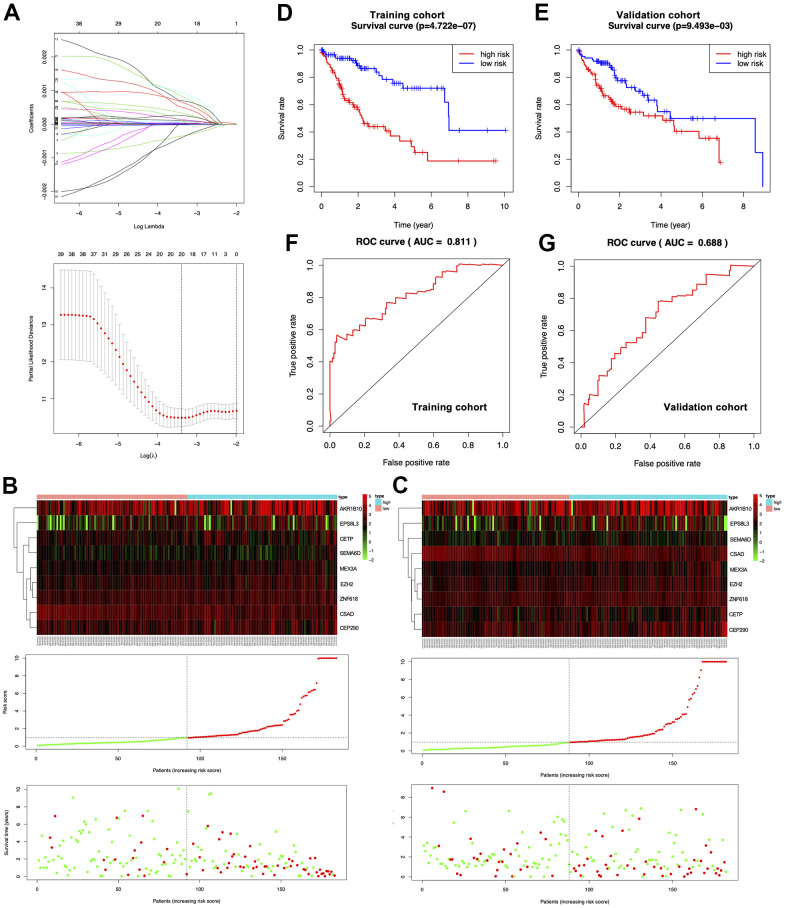
**Establishment of the prognosis prediction nomogram.** (**A**) Signature gene number determined through LASSO analysis. (**B**, **C**) The top, middle and bottom represent prognostic signature gene expression within the high- or low-risk group, risk score distribution, and patient survival of both cohorts, respectively. (**D**, **E**) KM curves for both cohorts. (**F**, **G**) ROC curves for both cohorts.

HCC data were obtained from the TCGA-LIHC cohort and randomly divided into training (n=183) and validation (n=182) cohorts. To identify the most representative genes associated with HCC survival from shared DEGs, we imported 633 genes by removing the overfitted genes using the LASSO regression algorithm and then analyzed them by multivariate Cox proportional risk regression (Figure 2A). Through this maneuver, we obtained nine risk genes from the training cohort to construct a prognostic model and determined the risk score of every specimen according to their expression and regression coefficients [25]. The median risk score in the training set was 0.9519; therefore, this value was selected for dividing specimens into high- and low-risk groups (Figure 2B center). The survival distribution in the training cohort is presented in Figure 2B (Figure 2B bottom). The variations in the expression of the nine risk genes in the heatmap conformed to the respective risk scores of the prognostic model (Figure 2B top). For the validation cohort, the median risk score was 0.9664. This study verified the prognostic prediction performance of the risk score and came to similar conclusions (Figure 2C). According to KM curve analysis, the high-risk group in the training cohort had a poorer prognosis than the low-risk group (Figure 2D; *p*=4.722e-07). Similarly, the high-risk group in the validation cohort had worse survival compared with the low-risk group (Figure 2E; *p*=9.493e-03). In addition, we drew ROCs and determined the AUC values of the two cohorts (0.811 and 0.688, respectively) (Figure 2F, 2G).

### Prioritization of candidate genes

**Figure 3:**
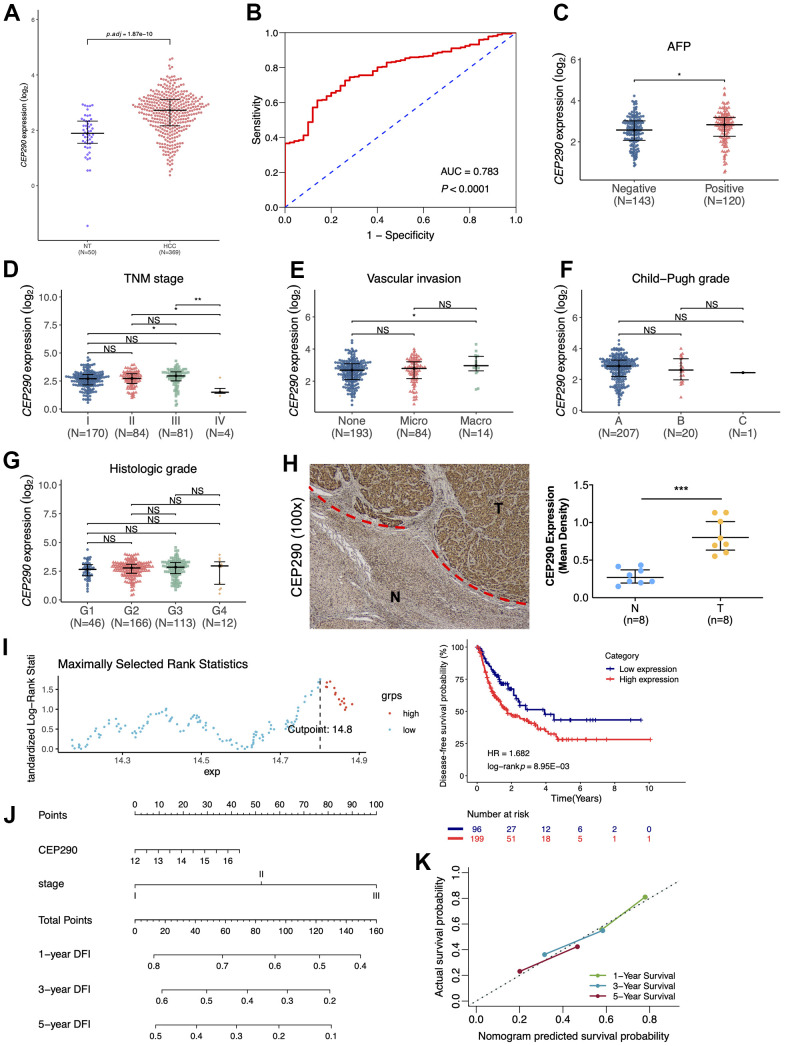
**Clinical significance of *CEP290*.** (**A**) The *CEP290* mRNA expression level is shown in TCGA-LIHC data (n=419). (**B**) Validation of the predictive value of *CEP290* upregulation in diagnosing HCC based on the ROC curve. (**C**–**G**) *CEP290* levels in comparison with AFP, TMN stage, vascular invasion, Child-Pugh grade and histologic grade. (**H**) Typical images (left) as well as quantification (right) of the IHC staining of *CEP290* in the 8 HCC specimens and matched non-cancerous specimens. (**I**) DFS analysis based on the X-tile plots threshold. (**J**) Prognosis nomogram for HCC cases after surgery. (**K**) Calibration curve for the nomogram used to predict 1-, 3- and 5-year DFS.

To better identify the most informative gene among those nine risk genes, we examined the signature genes for their clinical implications. According to primary screening, centrosomal protein 290 (*CEP290*), which was not reported in previous studies, has remarkable clinical value. *CEP290* was highly expressed in HCC specimens from the TCGA-LIHC cohort (n=419) (Figure 3A), and the ROC curve showed that upregulation of *CEP290* showed good diagnostic performance for HCC (Figure 3B; AUC=0.783; *p*<0.0001). Combined with other HCC clinical indicators, we found that *CEP290* overexpression was significantly related to TNM stage, vascular invasion and the alpha fetoprotein (AFP) level (Figure 3C–3E), whereas there was no significant difference in Child-Pugh grade or histologic grade (Figure 3F, 3G). Subsequently, we analyzed *CEP290* levels in eight HCC specimens and matched non-cancerous specimens. As suggested by the IHC assay, *CEP290* protein expression was significantly increased in HBV-associated HCC compared with matched non-cancerous specimens (Figure 3H). In addition, the prognostic value of *CEP290* for disease-free survival of HCC patients was evaluated by a KM curve analysis according to the best threshold (Figure 3I; cutoff point=14.8). We found that TCGA LIHC samples with *CEP290* overexpression had worse disease-free survival (DFS) (Figure 3I; HR=1.682, *p*=8.95e-03). To further validate the prognostic prediction performance of *CEP290* following radical hepatectomy, a nomogram was built base on TNM stage and *CEP290* level, the two independent factors for disease-free interval (DFI) discovered from multivariate analysis (Figure 3J). Clinicians were able to estimate 1-, 3- and 5-year disease-free survival based on the summation of each score from different prognostic factors incorporated in the nomogram. Calibration plots and bootstrap C-index were determined for the internal validation of the nomogram. Superior 1-, 3- and 5-year disease-free survival were estimated based on the bootstrapped calibration plots relative to the ideal model (Figure 3K). As a result, the above findings suggested that *CEP290* upregulation was a candidate biomarker to predict liver cancer prognosis.

**Figure 4:**
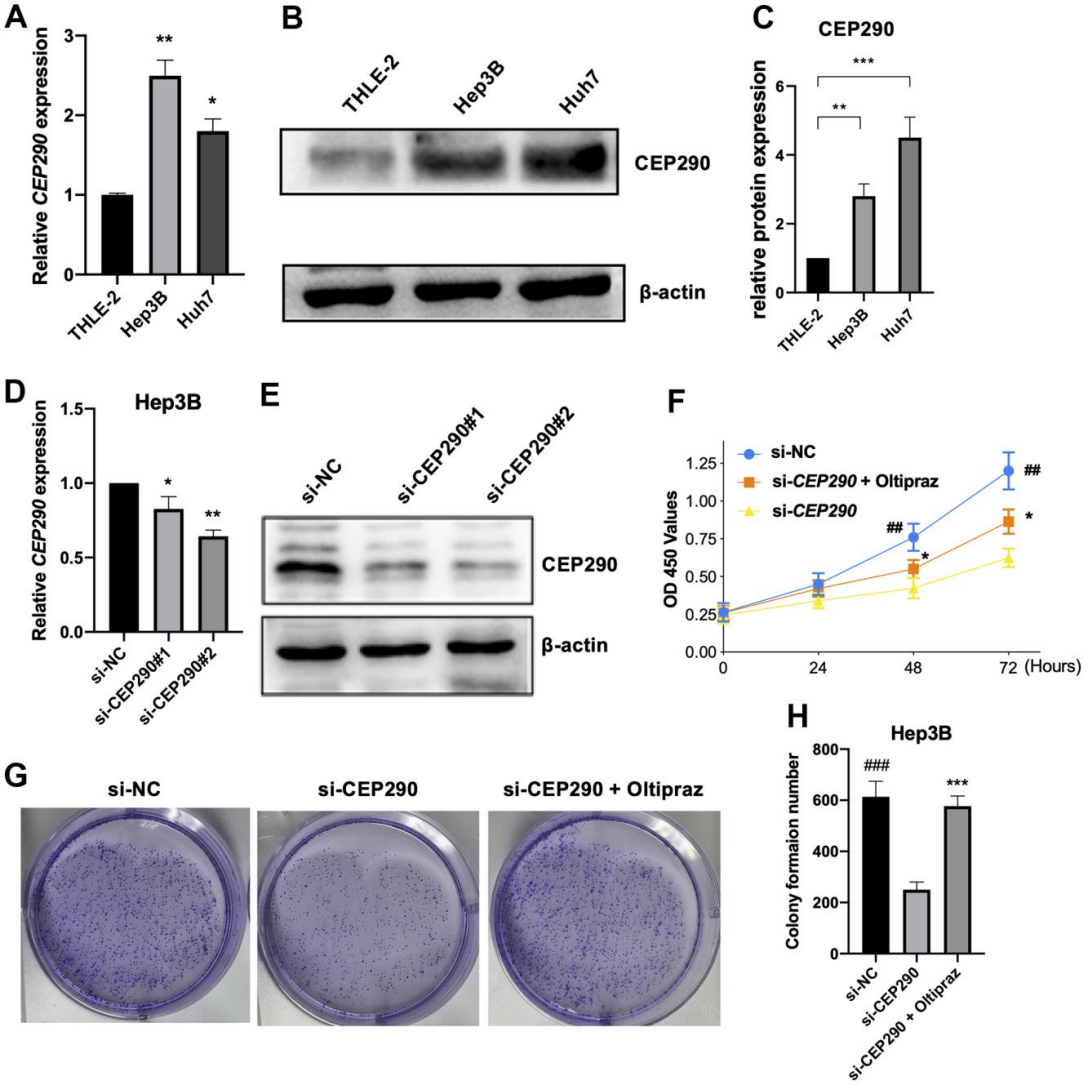
**Verification of *CEP290* expression levels in liver cancer cells.** (**A**, **B**) *CEP290* levels in HCC cells were measured by qRT–PCR and WB. (**C**) The quantification of protein expression of *CEP290* in diverse cell lines. (**D**, **E**) *CEP290* interference reduced mRNA and protein expression in Huh7 cells. (**F**) Cell proliferation measured by CCK-8 assay after *CEP290* interference. (**G**, **H**) Representative images (left) and quantification (right) of colony formation capacity. (*p < 0.05; **p < 0.01; ***p < 0.001; ##*p* < 0.01, ###*p* < 0.001).

We next extracted RNA for qRT-PCR and protein for WB from THLE-2, Huh7 and Hep3B cell lines. *CEP290* expression was elevated in Hep3B and Huh7 cell lines relative to THLE-2 cells when examined by qRT–PCR and WB (Figure 4A–4C). These results suggested *CEP290* might play an important role in liver cancer. Although *CEP290* mRNA was more abundantly expressed in Hep3B cells compared with Huh7 cells, the protein expression in Hep3B cells was lower than that in Huh7 cells. As a result, we chose Hep3B cells for subsequent experiments.

### *CEP290* knockdown suppressed liver cancer cell malignant phenotypes and ferroptosis

**Figure 5:**
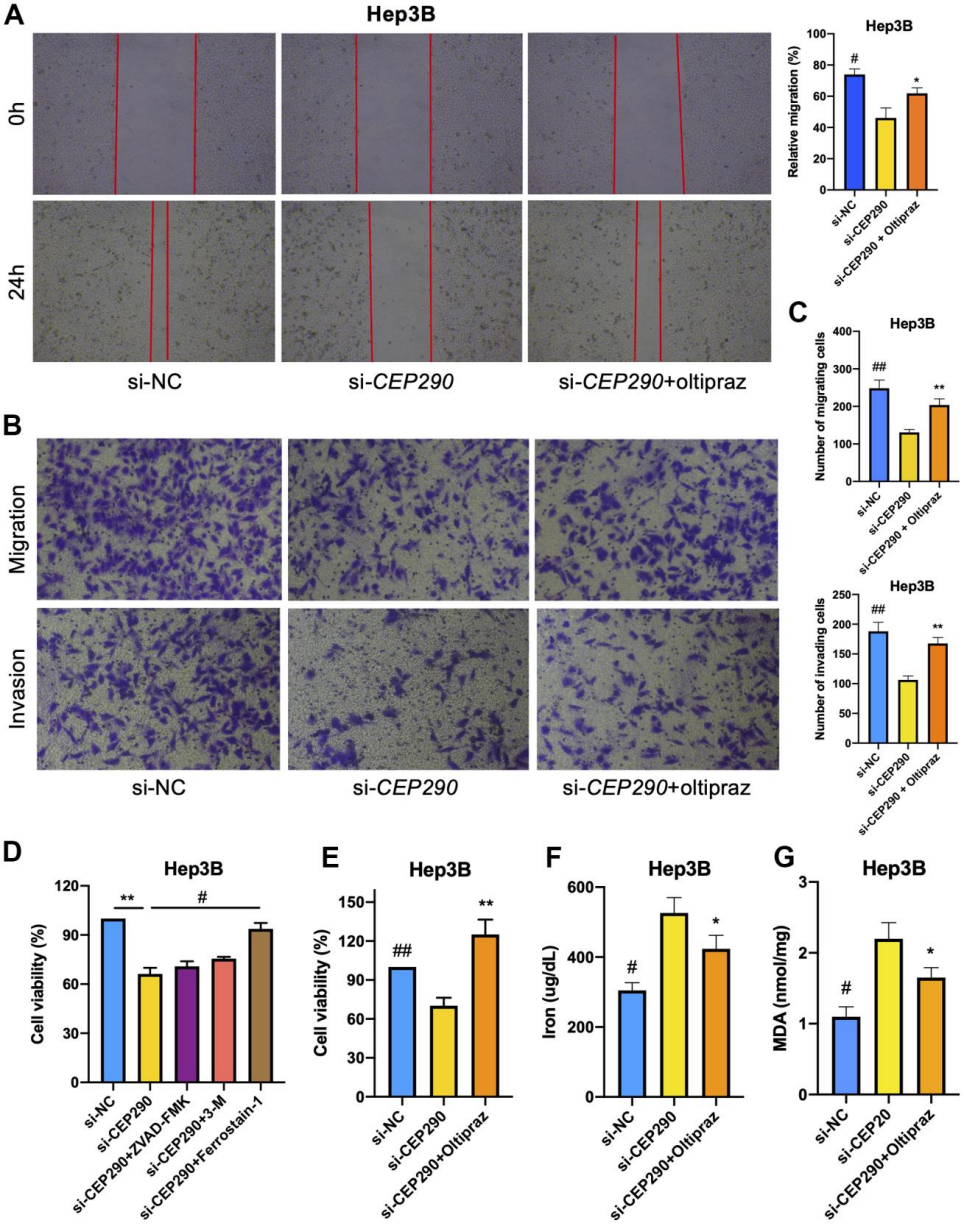
**Effect of *CEP290* interference on liver cancer cell ferroptosis and malignant phenotypes.** (**A**) Scratch assay. Scratches were made in a confluent culture, and cell migration into the scratch wounds was documented. (**B**, **C**) Transwell assays. After treatment, the migration and invasion of Hep3B cells were analyzed. (**D**) Hep3B cell viability was evaluated after si-*CEP290*, incorporate with treatment of Z-DEVD-FMK (100 μM), 3-MA (10 mM), and ferrostain-1 (100 μM). (**E**–**G**) Cell viability, ferrous iron and MDA levels in Hep3B cells treated with si-*CEP290* and 75 μM oltipraz (* si-*CEP290* vs. si-*CEP290*+oltipraz, **p < 0.01, *p < 0.05; # si-NC vs. si-*CEP290*, ##*p* < 0.01, #*p* < 0.05).

To elucidate the role of *CEP290* in HCC, we transfected Hep3B cells with small interfering RNA (siRNA) specific to *CEP290* (siRNA #1 and #2). *CEP290* mRNA levels were markedly reduced in Hep3B cells transfected with si-*CEP290*#1 and #2, indicating that *CEP290* was successfully silenced (Figure 4D, 4E). Si-*CEP290* exhibited superior activity and was used for subsequent analysis. Compared to NC-siRNA, Hep3B cells transfected with *CEP290*-siRNA showed significantly decreased proliferation (Figure 4F). In addition, a colony formation assay was detected. *CEP290* inhibition reduced the number of colonies formed (Figure 4G, 4H). Furthermore, *CEP290*-silenced cells exhibited decreased migration and invasion compared with NC-siRNA cells (Figure 5A–5C). Therefore, we concluded that *CEP290* played an essential role in the invasion and migration of Hep3B cells.

Next, to determine the mode of cell death, we treated Hep3B cells with the apoptosis inhibitor Z-DEVD-FMK, autophagy inhibitor 3-MA, and ferroptosis inhibitor ferrostain-1. The results demonstrated that both apoptosis and autophagy inhibition had only marginal impact on the cell death induced by *CEP290* depletion. However, the ferroptosis inhibitor strikingly reduced melanoma cell death induced by the suppression of *CEP290* (Figure 5D). The above results suggested that *CEP290* inhibition promoted ferroptosis in liver cancer cells (Figure 5E). Subsequently, we evaluated ferroptosis-related markers such as lipid peroxidation (MDA) and iron accumulation upon *CEP290* interference to determine the association of *CEP290* with ferroptosis in Hep3B cells. *CEP290* silencing increased the intracellular Fe^2+^ level (Figure 5F). Furthermore, the accumulation of MDA, the representative end product of lipid peroxidation, was analyzed in Hep3B cells depleted of *CEP290*. *CEP290* inhibition enhanced MDA accumulation in Hep3B cells (Figure 5G). The above findings suggested a possible role of *CEP290* as a ferroptosis regulating factor that modulates MDA and Fe^2+^ contents in Hep3B cells.

### *CEP290* knockdown inhibited *Nrf2* signaling pathway

**Figure 6:**
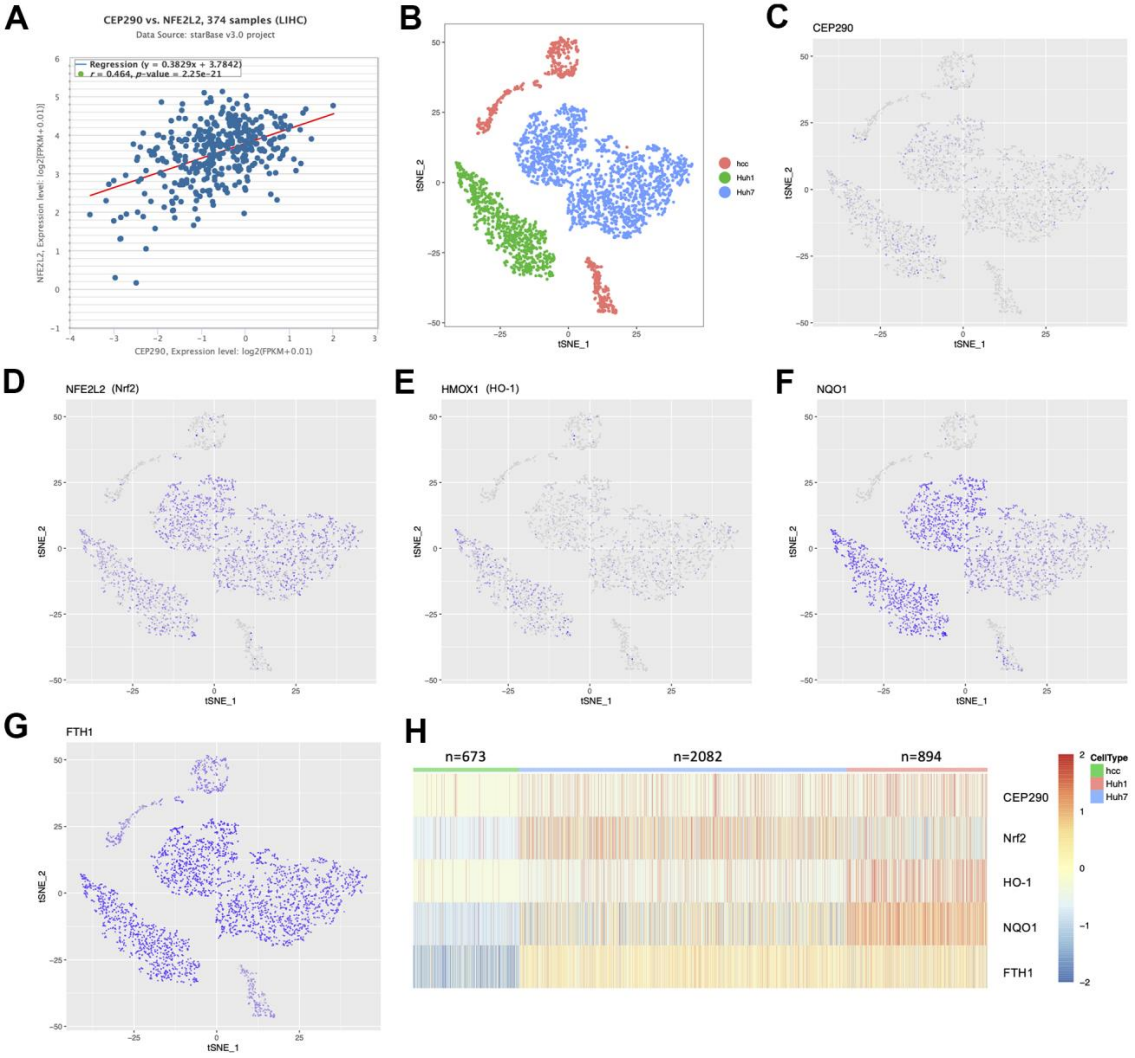
**The expression distribution between *CEP290* and *Nrf2* pathway members in single-cell profiling.** (**A**) Co-expression correlation analysis using the TCGA-LIHC RNA-Seq cohort (n=374). (**B**) t-SNE map shows three liver cancer cell classes. Each dot represents one cell. (**C**–**H**) Profile of target expression (scRNA-Seq) based on the t-SNE plot from Figure 6B.

Enrichment analysis suggested that (Figure 1F) the oxidant-stress response played an important role in HCC development. Therefore, we conducted correlation analysis of the TCGA-LIHC RNA-Seq cohort (n=374) to investigate the relationship of *CEP290* with the oxidant-stress core gene *Nrf2*. *CEP290* expression showed a positive correlation with *Nrf2* (Figure 6A; r=0.464, *p*=2.25e-21), which suggests *CEP290* may be functionally related to the *Nrf2*-mediated oxidant stress response. Next, we sought to determine the expression landscape of *CEP290* and the genes in the *Nrf2* signal transduction pathway in HCC cells using 10×Genomics single-cell RNA sequencing data (GSE103867) [18]. The tSNE clustering analysis clearly distinguished between resected HCC (hcc), Huh1 and Huh7 cells (Figure 6B). However, *CEP290*, *HO-1*, *Nrf2*, *NQO1* and *FTH1* levels were basically consistent (Figure 6C–6H), implying that the *Nrf2* pathway activation may be associated with *CEP290* overexpression.

**Figure 7:**
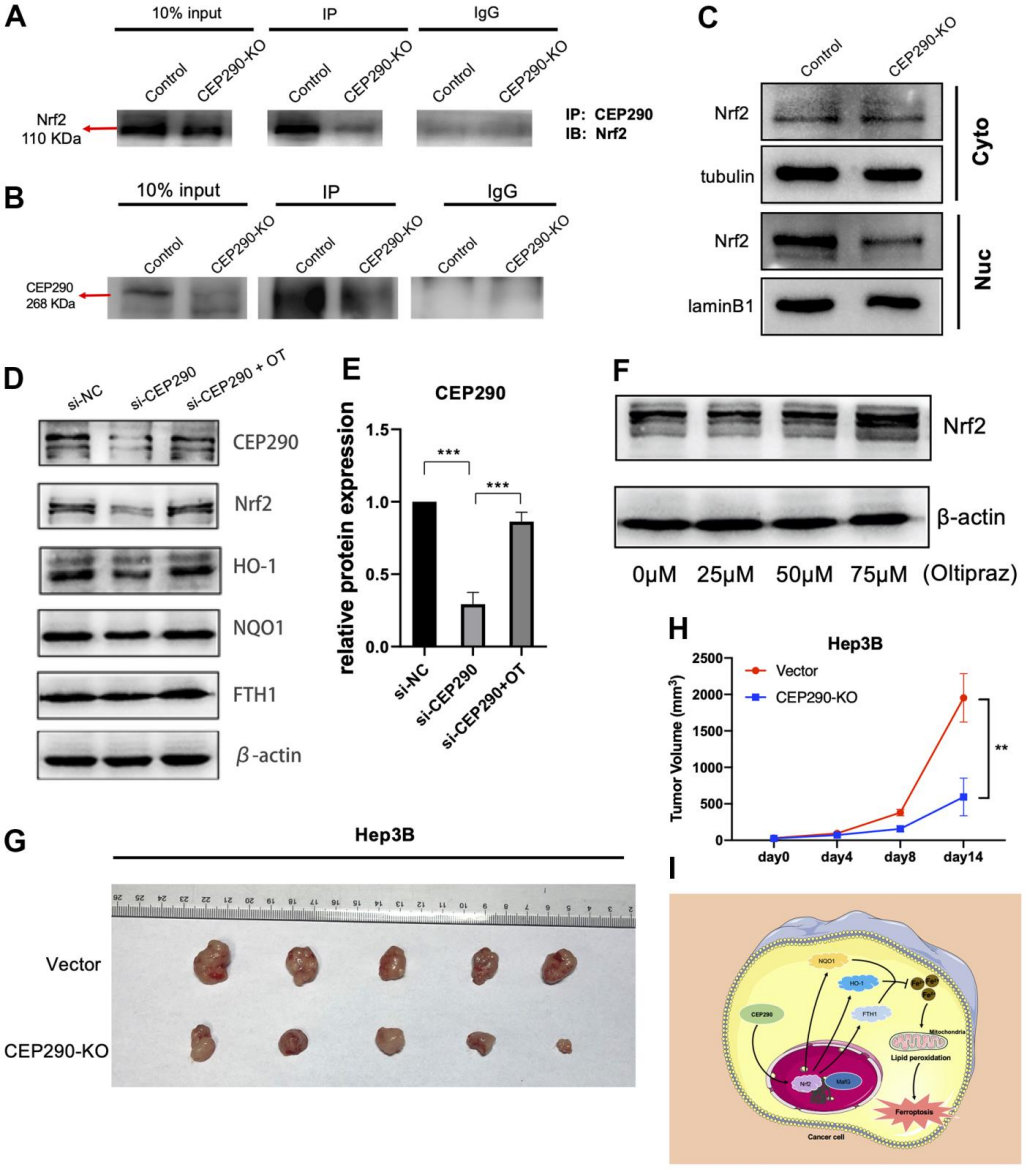
***CEP290* knockout inhibits tumor growth *in vivo*.** (**A**, **B**) The interaction between *CEP290* and *Nrf2* was analyzed by WB and immunoprecipitation in Hep3B cells. (**C**) *Nrf2* expression in nuclear extracts after *CEP290* knockout. (**D**) Following *CEP290* silencing, the protein expression of *Nrf2* and the respective downstream members (*HO-1*, *FTH1* and *NQO1*) were detected by WB, and (**E**) the protein levels of *CEP290* were quantified. (**F**) Selection of optimal oltipraz dose. (**G**) Gross appearances of tumors. Male nude mice (six weeks old, n = 5 for each group) were subcutaneously injected with Hep3B and *CEP290*-KO Hep3B cells (2 × 10^6^) on the right flank. The mouse tumors were collected 2 weeks after implantation. (**H**) Xenograft tumor sizes were determined in mice bearing tumors. (**I**) A proposed model of the *CEP290*/*Nrf2* axis activation in HCC. All results are presented as the mean ± SD; **p < 0.01; ***p < 0.001.

Then, we explored the potential mechanism how *CEP290* might regulate *Nrf2* expression. A previous article reported that *Nrf2* is a vital transcription factor [26]; hence, we hypothesized that *CEP290* may interact with *Nrf2*, promote *Nrf2* transcription and activate the *Nrf2* signaling pathway. To test this, we first evaluated the *CEP290*-*Nrf2* interaction within Hep3B cells by conducting CoIP assays. Since the siRNA approach may not be sufficient to show the effect of *CEP290* depletion in CoIP experiments clearly, we generated a cell line (Hep3B *CEP290*^-/-^) with a homozygous knockout of *CEP290* using the CRISPR/Case9 gene editing system. CoIP results confirmed the interaction between *CEP290* and *Nrf2* (Figure 7A, 7B). Western blot results showed that nuclear translocation of *Nrf2* in *CEP290*-KO Hep3B cells was reduced (Figure 6C), and *CEP290* silencing significantly downregulated the expression levels of *Nrf2* signaling pathway members (*FTH1*, *Nrf2*, *NQO1*, *HO-1*) (Figure 7D, 7E). These observations demonstrated that *CEP290* promoted the transcription of *Nrf2* pathway genes through *Nrf2*.

We next used oltipraz, an activator of *Nrf2*, to determine whether the inhibitory effect of *CEP290* could be circumvented by *Nrf2* activation. Hep3B cells were treated at different concentrations to determine an appropriate dose, and we found that 75 μM oltipraz had the optimal effect as shown in Figure 7F. Administration of oltipraz completely reversed the effect of *CEP290* silencing on cell growth, migration, invasion and ferroptosis (Figures 4F–4H, 5A–5C, 5E–5G). Furthermore, oltipraz treatment upregulated the expression levels of the *Nrf2* pathway genes (*Nrf2*, *HO-1, NQO1* and *FTH1*) (Figure 7D, 7E). These findings suggested that *CEP290* regulated biological behaviors and ferroptosis in HCC cells by activating the *Nrf2* pathway.

### Loss of *CEP290* suppresses tumor growth *in vivo*

Since *CEP290* silencing suppressed HCC malignant behaviors *in vitro*, we investigated whether *CEP290* knockout inhibited tumor growth *in vivo*. *CEP290* knockout (*CEP290*-KO) Hep3B cells (2×10^6^ per injection site) were injected into nude mice subcutaneously. Compared to the control group, *CEP290*-KO cells formed smaller tumors (Figure 7G, 7H). Collectively, these results suggest that *CEP290* plays an important role in the regulation of HCC proliferation in a subcutaneous xenograft model.

## DISCUSSION

In the present work, we downloaded gene expression profile datasets GSE54238 and GSE22058 and identified 633 shared DEGs. Through a series of bioinformatic analyses and *in vitro* and *in vivo* assays, we identified *CEP290* as a novel candidate biomarker of HCC prognosis, demonstrated that CEP290 played important functions in the growth, migration, invasion and ferroptosis of HCC cells and explored its potential mechanism of action. This study provides strong evidence that *CEP290* functions in regulating ferroptosis in liver cancer cells via the *Nrf2* signaling pathway (Figure 7I).

Ferroptosis is a novel iron-dependent form of cell death f [27] and is regulated by diverse signal transduction pathways in cancers [28]. *SLC7A11* regulated cancer cell ferroptosis through glucose- and glutamine-dependency [29]. *GLRX5* knockdown stimulated the iron-starvation response and increased intracellular Fe^2+^ through iron-responsive element-binding protein, thus inducing ferroptosis [30]. However, the regulatory mechanism of ferroptosis remains ambiguous.

As evident from recent findings, centrosomal protein (*CEP*) plays an important role in carcinogenesis. For example, *CEP55* overexpression increases cancer cell stemness and enhances tumor formation by activating the PI3K/AKT pathway [31]. *TACC3*, a key centrosomal protein, was upregulated in prostate cancer, and its silencing inhibited tumor growth [32]. *CEP290* was previously reported to be involved in cell ciliogenesis [33]. Deletion of *CEP290* blocked the formation of cilia by directly recruiting *DAZ* and zinc finger protein 1 *DZIP1* [34] or by disrupting the formation and subcellular distribution of the protein complex *PCM-1* [35]. However, the biological function of *CEP290* is virtually unexplored in cancers. In our study, *CEP290* was identified from a prognostic signature screening. It is overexpressed in the TCGA-LIHC cohort, HCC tissues and liver cancer cell lines. Using multivariate analyses, we found that *CEP290* expression in TCGA-LIHC data could serve as an independent indicator of poor prognosis. In addition, overexpression of *CEP290* was also correlated with the AFP level, TNM stage and vascular invasion, suggesting that *CEP290* could be used as a prognostic marker.

To clarify the contribution of *CEP290* to liver cancer progression, we examined the effect of *CEP290* depletion both *in vitro* and *in vivo*. In Hep3B cells, the suppression of *CEP290* expression inhibited cell proliferation, migration and invasion and induced ferroptosis. Moreover, *CEP290* knockout repressed Hep3B cell tumor formation *in vivo*. These findings proved that *CEP290* contributed to hepatocarcinogenesis and key implication for the development of therapeutic tactics of HCC.

*Nrf2*, a core gene for the oxidant stress response, activates its downstream genes and would promote cancer progression [36], and therefore, *Nrf2* is a candidate therapeutic target to treat cancer [37, 38]. According to published literature, *Nrf2* knockdown inhibited HCC development [39, 40], which suggested that *Nrf2* induces HCC by activating downstream targets. Our results have confirmed the interaction between *CEP290* and *Nrf2* proteins. *CEP290* silencing blocked the nuclear translocation of *Nrf2* and reduced the expression of *Nrf2* pathway members. Administration of *Nrf2* activator oltipraz elevated the expression of *Nrf2* pathway members and induced malignant phenotypes of liver cancer cells in *CEP290*-depleted cancer cells. These data revealed that *CEP290* modulated ferroptosis and malignant phenotypes of liver cancer cells by regulating the *Nrf2* signaling pathway.

## CONCLUSIONS

In summary, the present work established DEGs from 2 independent HCC datasets and identified *CEP290* as a predictor of patient survival. *CEP290* is involved in the ferroptosis of HCC cells via the *Nrf2* pathway. The present study provides a novel direction in deciphering the mechanism of HCC initiation.
